# Rapid evolutionary divergence of diploid and allotetraploid *Gossypium* mitochondrial genomes

**DOI:** 10.1186/s12864-017-4282-5

**Published:** 2017-11-13

**Authors:** Zhiwen Chen, Hushuai Nie, Yumei Wang, Haili Pei, Shuangshuang Li, Lida Zhang, Jinping Hua

**Affiliations:** 1 Laboratory of Cotton Genetics, Genomics and Breeding /Key Laboratory of Crop Heterosis and Utilization of Ministry of Education/Beijing Key Laboratory of Crop Genetic Improvement, College of Agronomy and Biotechnology, China Agricultural University, Beijing, 100193 China; 2 Institute of Cash Crops, Hubei Academy of Agricultural Sciences, Wuhan, Hubei 430064 China; 3 Department of Plant Science, School of Agriculture and Biology, Shanghai Jiao Tong University, Shanghai, 200240 China

**Keywords:** Mitochondrial genomes, Comparative genomics, Multiple DNA rearrangement, Unique segments, Repeat sequences, *Gossypium*

## Abstract

**Background:**

Cotton (*Gossypium* spp.) is commonly grouped into eight diploid genomic groups and an allotetraploid genomic group, AD. The mitochondrial genomes supply new information to understand both the evolution process and the mechanism of cytoplasmic male sterility. Based on previously released mitochondrial genomes of *G. hirsutum* (AD_1_), *G. barbadense* (AD_2_), *G. raimondii* (D_5_) and *G. arboreum* (A_2_), together with data of six other mitochondrial genomes, to elucidate the evolution and diversity of mitochondrial genomes within *Gossypium*.

**Results:**

Six *Gossypium* mitochondrial genomes, including three diploid species from D and three allotetraploid species from AD genome groups (*G. thurberi* D_1_, *G. davidsonii* D_3-d_ and *G. trilobum* D_8_; *G. tomentosum* AD_3_, *G. mustelinum* AD_4_ and *G. darwinii* AD_5_), were assembled as the single circular molecules of lengths about 644 kb in diploid species and 677 kb in allotetraploid species, respectively. The genomic structures of mitochondrial in D group species were identical but differed from the mitogenome of *G. arboreum* (A_2_), as well as from the mitogenomes of five species of the AD group. There mainly existed four or six large repeats in the mitogenomes of the A + AD or D group species, respectively. These variations in repeat sequences caused the major inversions and translocations within the mitochondrial genome. The mitochondrial genome complexity in *Gossypium* presented eight unique segments in D group species, three specific fragments in A + AD group species and a large segment (more than 11 kb) in diploid species. These insertions or deletions were most probably generated from crossovers between repetitive or homologous regions. Unlike the highly variable genome structure, evolutionary distance of mitochondrial genes was 1/6th the frequency of that in chloroplast genes of *Gossypium*. RNA editing events were conserved in cotton mitochondrial genes. We confirmed two near full length of the integration of the mitochondrial genome into chromosome 1 of *G. raimondii* and chromosome A03 of *G. hirsutum*, respectively, with insertion time less than 1.03 MYA.

**Conclusion:**

Ten *Gossypium* mitochondrial sequences highlight the insights to the evolution of cotton mitogenomes.

**Electronic supplementary material:**

The online version of this article (10.1186/s12864-017-4282-5) contains supplementary material, which is available to authorized users.

## Background

Plant mitochondrial genomes (mtDNA) embrace notable characteristics, such as an extreme and highly diverse mitochondrial genome structure [1–4]. Plant mitochondrial genomes also possess highly branched and sigma-like structures [5–7] as well as multichromosomal genomes recently identified in three distantly-related angiosperm lineages [8–10]. The mitochondrial genome in plants is also noteworthy in that there is large variation in genome size (ranging from ∼66 kb to 11.3 Mb) [8, 11] with highly variable intergenetic regions and a considerable proportion of repeated sequences [12], frequent rearrangements [13], massive gene loss [14], and frequent endogenous and foreign DNA transfer [15–17].

In terms of structure, angiosperm mitochondrial genomes are typically mapped as circular molecules with one or more larger (>1 kb) repetitive sequences, which promote active homologous inter- and intra-genomic recombination [4, 18, 19]. However, it is not clear how plant mitochondrial genomes rearrange so frequently or how the genome sizes can vary dramatically over relatively short evolutionary period. This dynamic organization of the angiosperm mitochondrial genome provides unique information as well as an appropriate model system for studying genome structure and evolution. More syntenic sequences will be helpful to interpret the evolutionary processes for diverse angiosperm mitochondrial structures.

Cotton (*Gossypium*) is the most important fiber crop plant in the world [20]. Four domesticated species remain as cultivated crops: the New World allopolyploid species *G. hirsutum* and *G. barbadense* (2n = 52), and the Old World diploid species *G. arboreum* and *G. herbaceum* (2n = 26) [20, 21]. The primary cultivated one is Upland cotton (*G. hirsutum* L.), accounting for more than 90% of global cotton fiber output. *Gossypium* includes 52 species: seven allotetraploid species and 45 diploids [21, 22]. The nascent allopolyploid species spread throughout the American tropics and subtropics, diverging into at least seven species, namely, *G. hirsutum* L. (AD_1_), *G. barbadense* L. (AD_2_), *G. tomentosum* Nuttalex Seemann (AD_3_), *G. mustelinum* Miersex Watt (AD_4_), *G. darwinii* Watt (AD_5_), *G. ekmanianum* (AD_6_), and *G. stephensii* (AD_7_) [20–22]. The diploid *Gossypium* species comprise eight monophyletic genome groups, A, B, C, D, E, F, G and K group [20, 23]. With the rapid development of next-generation sequencing technologies [24, 25], cotton genomics research has rapidly progressed in recent years, such that nuclear genome sequences have now been published for model diploids (D_5_-genome [26, 27], A_2_-genome [28]), and for the allopolyploids (AD_1_-*G. hirsutum* [29, 30], AD_2_-*G. barbadense* [31, 32]). In addition, a large number of *Gossypium* organelle genome sequences have been released [33–39]. Compared to the highly conserved chloroplast genome structures [34–36], comparative analysis revealed rapid evolutionary divergence of *Gossypium* mitochondrial genomes [37–39], which proved that deep analyses of more mitochondrial genomes would provide new data to consider the evolutionary relationships and to explore the mechanism of cytoplasmic male sterility (CMS).

Cytoplasmic male sterility (CMS) is a maternally-conferred reproductive trait that relies on the expression of CMS-inducing mitochondrial sequences [40]. Many examples of CMS stem from the consequences of recombination [40–42]. Often, these chimeric CMS genes exhibit co-transcription with upstream or downstream functional genes, which typically affect the mitochondrial electron transfer chain pathways to fail to produce functional pollen [43]. Rearrangements in the mitochondrial DNA involving known mitochondrial genes as well as unknown sequences result in the creation of new chimeric open reading frames, which encode proteins containing transmembrane and lead to cytoplasmic male sterility by interacting with nuclear-encoded genes [43–45].

Here, six *Gossypium* mitochondrial genomes are reported, including three diploid species from D genome groups (*G. thurberi* D_1_, *G. davidsonii* D_3-d_ and *G. trilobum* D_8_) and three allotetraploid species from AD genome groups (*G. tomentosum* AD_3_, *G. mustelinum* AD_4_ and *G. darwinii* AD_5_). Comparative mitochondrial genome analysis then revealed rapid mitochondrial genome rearrangement and evolution between diploid and allotetraploid *Gossypium*. In addition, one of the most surprising outcomes of comparative analyses is how rapidly mitochondrial sequence segments altered within a single subspecies. Finally, the four mitogenomes of D group species provided the useful data resources for interpreting the CMS-related genes in *G. trilobum* D_8_ cotton.

## Methods

### Plant materials and mitochondrial DNA extraction

Seeds of diploid and allotetraploid *Gossypium* species were acquired from the nursery on the China National Wild Cotton Plantation in Sanya, Hainan, China. Mitochondria were isolated from week-old etiolated seedlings, and the mitochondrial DNA samples were extracted from an organelle-enriched fraction isolated by differential and sucrose gradient centrifugation, essentially as described earlier [37–39, 46].

### Mitochondrial genome sequencing and primary data processing

A total of ~5 million clean paired-end reads were sequenced from a ~500 bp library for each of three diploid species, respectively. We produced 300 bp read length with paired-end sequencing, using MiSeq sequencing method on Illumina platform at Beijing Biomarker Technologies Co, LTD. A total of ~11 million clean paired-end reads were sequenced from a ~500 bp library with paired-end, 300 bp read length, for each of three allotetraploid species, respectively, using the same method. Raw sequences were first evaluated by two quality control tools, Trimmomatic [47] and FilterReads module in Kmernator ([https://github.com/JGI-Bioinformatics/Kmernator]) to remove any potential undesirable artifacts in the data such as adapters or low quality or “N” bases and so on.

### Genomes assembly and sequence verification

Six *Gossypium* draft mitogenomes were assembled de novo from the clean reads with velvet 1.2.10 [48] or combining FLASH [49] and Newbler (Version 2.53) methods, respectively. For the first assembly method, i.e., the 300-bp paired-end reads from six *Gossypium* species, we performed multiple velvet runs with different combinations of kmer values (for (kmer = 75; kmer <=209; kmer = kmer +2), (42 in total)). Three Kmer values (193, 195, 197), owning larger N50 values, less contig number, were used to assemble the mitogenomes. For each velvet run, the minimum coverage parameter was set to 10× and scaffolding was turned off when the data sets contained paired-end reads. For each of assembly, mitochondrial contigs were identified by blastn [50] searches with known *Gossypium* mitochondrial genomes for scaffolding and gap filling [37–39]. The best draft assemblies for six *Gossypium* were chosen as the assembly that maximized total length of mitochondrial contigs after combining three Kmer values assembly. In another assembly method, we combined FLASH [49] and Newbler (Version 2.53) softwares together. First, FLASH provides the use of paired-end libraries with a fragment size (500 bp) shorter than twice the read length (300 bp) an opportunity to generate much longer reads (500 bp) by overlapping and merging read pairs [49]. The merging file was then assembled using Newbler (Version 2.53) software. Finally, the assembled mitochondrial scaffolds were aligned with known *Gossypium* mitochondrial genomes [37–39] for anchoring scaffold directions and gap filling. Thus, we combined two types of the assembly results to complete the six *Gossypium* mitogenomes. The final remaining gaps were filled by aligning individual pair-end sequence reads that overlapped the scaffolds or contig ends using Burrows-Wheeler Aligner (BWA 0.7.10-r789) software [51].

To evaluate six mitogenomes sequence assembly quality and accuracy, pair-end reads were mapped onto their respective consensus sequences with BWA 0.7.10-r789 [51]. The BWA mapping resulting SAM files were transformed into BAM files using samtools view program [52]. The BWA mapping results for these pair-end reads in BAM files were then used to calculate depth of sequencing coverage through samtools depth program [52]. For all six *Gossypium* species, the Illumina reads covered all parts of the genome consistently, with the average coverage ranging from 50× to 200 × .

### Genome annotations and sequence analyses

*Gossypium* mitochondrial genes from the six species were annotated using *G. hirsutum* and *G. barbadense* mitogenomes as references. Functional genes (other than tRNA genes) were identified by local blast searches against the database, whereas tRNA genes were predicted de novo using tRNA scan-SE [53]. Repeat-match program in MUMmer [54] was used to identify repeated sequences within six *Gossypium* mitogenomes. Their genome maps were generated using OGDRAW [55] and the repeat map was drawn by Circos [56].

Collinear blocks were generated among the ten mitochondrial genomes of *Gossypium* using the progressiveMauve program [57]. To determine the amount of *Gossypium* mitochondrial genome complexity shared between species, each pair of mitogenomes was aligned using blastn [50] with an e-value cutoff of 1 × 10^−5^. Using these parameters, the blastn searches should be able to detect homologous sequences as short as 30 bp. The unique segments in *Gossypium* mitogenomes identified in this study were summarized as follows: i) Paired-end reads were mapped onto their respective consensus sequences using the Burrows-Wheeler Aligner (BWA 0.7.10-r789) software [51]; ii) The BWA mapping resulting SAM files were transformed into BAM files using the samtools program [52] set to the default parameters; and iii) Structure variations (SVs) and InDels reported in this work were manually visualized using the Integrative Genomics Viewer (IGV) software [58].

### RNA editing identification

RNA edit sites were computationally predicted using the batch version of the PREP-Mt. online server [59], with a cutoff value of 0.2.

### Phylogenetic analyses and estimation of evolutionary divergence

For phylogenetic analyses, 36 protein-coding genes were extracted from 10 *Gossypium* species and two outgroups: *C. papaya* and *A. thaliana*. Sequence alignments for 36 concatenated genes, each chloroplast and mitochondrial coding exons were carried out by MAFFT [60]. Phylogenetic analyses were performed with the same methods to our previous studies [35, 36, 39]. P-distances for chloroplast and mitochondrial coding genes were calculated with MEGA5.05 [61].

### Identifying nuclear mtDNAs in *Gossypium*, estimation of evolutionary divergence and divergence time between mitochondrial sequences and *numts*

Dot matrix comparisons were generated between the mitochondrial and nuclear chromosomes of four *Gossypium* species using the nucmer program of MUMmer with the parameters 100-bp minimal size for exact match and 500-bp minimal interval between every two matches [54]. The detailed comparison results were shown in Fig. 7: *G. raimondii* mitochondrial [39] and nuclear chromosomes [27] in Fig. 7a, *G. arboreum* mitochondrial [39] and nuclear chromosomes [28] in Fig. 7b, *G. hirsutum* mitochondrial [37] and nuclear chromosomes [30] in Fig. 7c, *G. barbadense* mitochondrial [38] and nuclear chromosomes [32] in Fig. 7d. Sequence alignments for each coding, intronic, and intergenic spacer regions were carried out by MAFFT [60] software. P-distances between mitochondrial sequences and numts were calculated with MEGA5.05 [61]. In order to estimate how old these insertions are, p-distance rates and some estimates of rate/million years were studied here. The divergence time of between mitochondrial native sequences and numts was calculated by the following Formula: T = p-distance/(r_nu_ + r_mt_) [62]. Based on Gaut et al. (1996) and Muse et al. (2000), the r_nu_ and r_mt_values were estimate as r_nu_ = 6.5 × 10^−9^ and r_mt_ = 2 × 10^−10^, respectively [63, 64]. It also has to be made clear that the underlying assumption is homogeneity in rate since their divergence from a common ancestor.

## Results

### *Gossypium* mitochondrial genomes from diploid and allotetraploid species

**Fig. 1:**
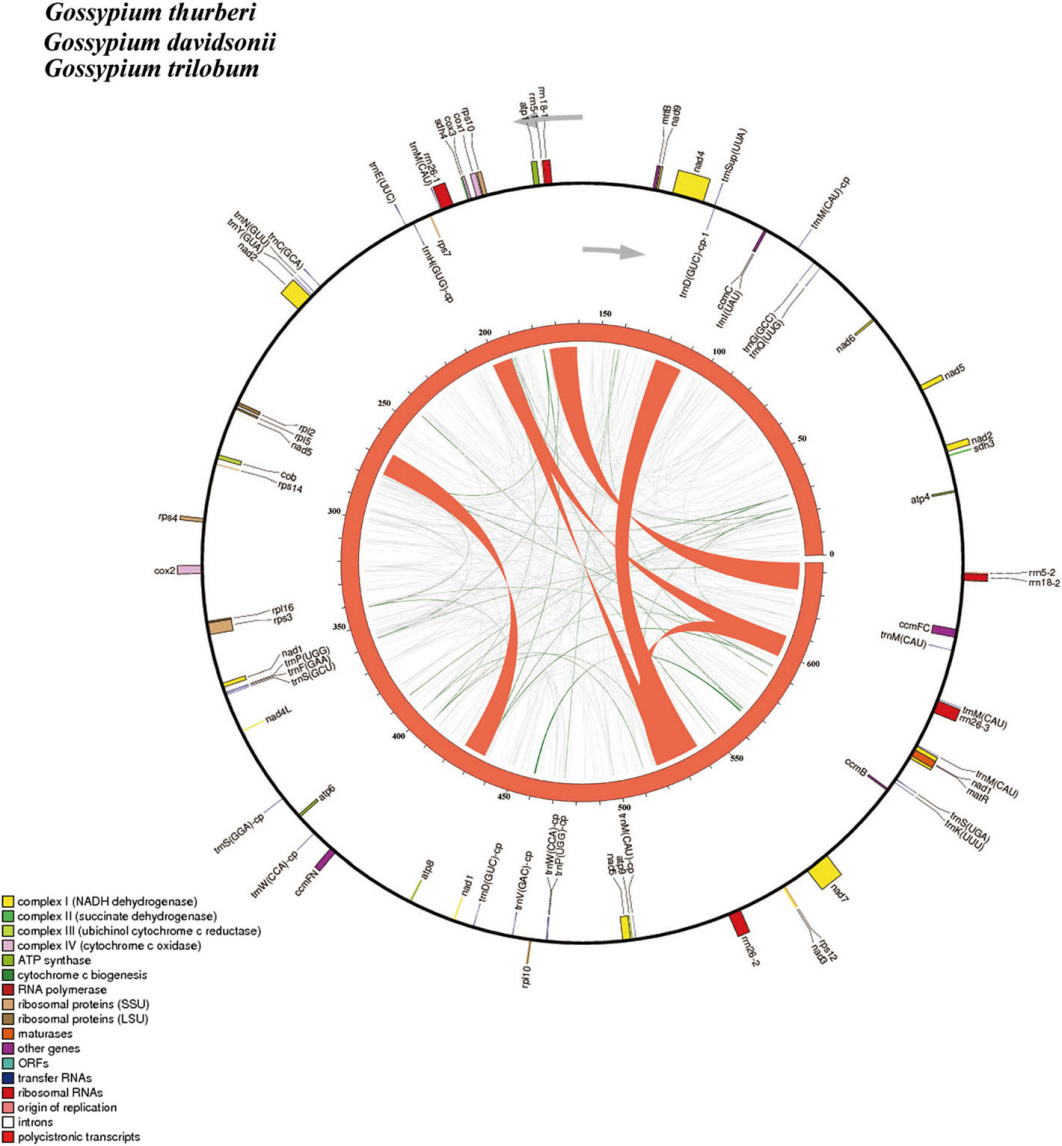
Genome maps of three diploid *Gossypium* mitogenomes. The map shows both the gene map (outer circle) and repeat map (inner map). Genes exhibited on the inside of outer circles are transcribed in a clockwise direction, while genes on the outside of outer circles are transcribed in a reverse direction. The inner circle reveals the distribution of repeats in two mitogenomes with curved lines and ribbons connecting pairs of repeats and width proportional to repeat size. The red ribbons represent > = 1 Kb repeats, the very deep green lines represent repeats between 100 bp to 1 Kb and the very light grey lines represent repeats <100 bp. The numbers give genome coordinates in kilobase

**Fig. 2:**
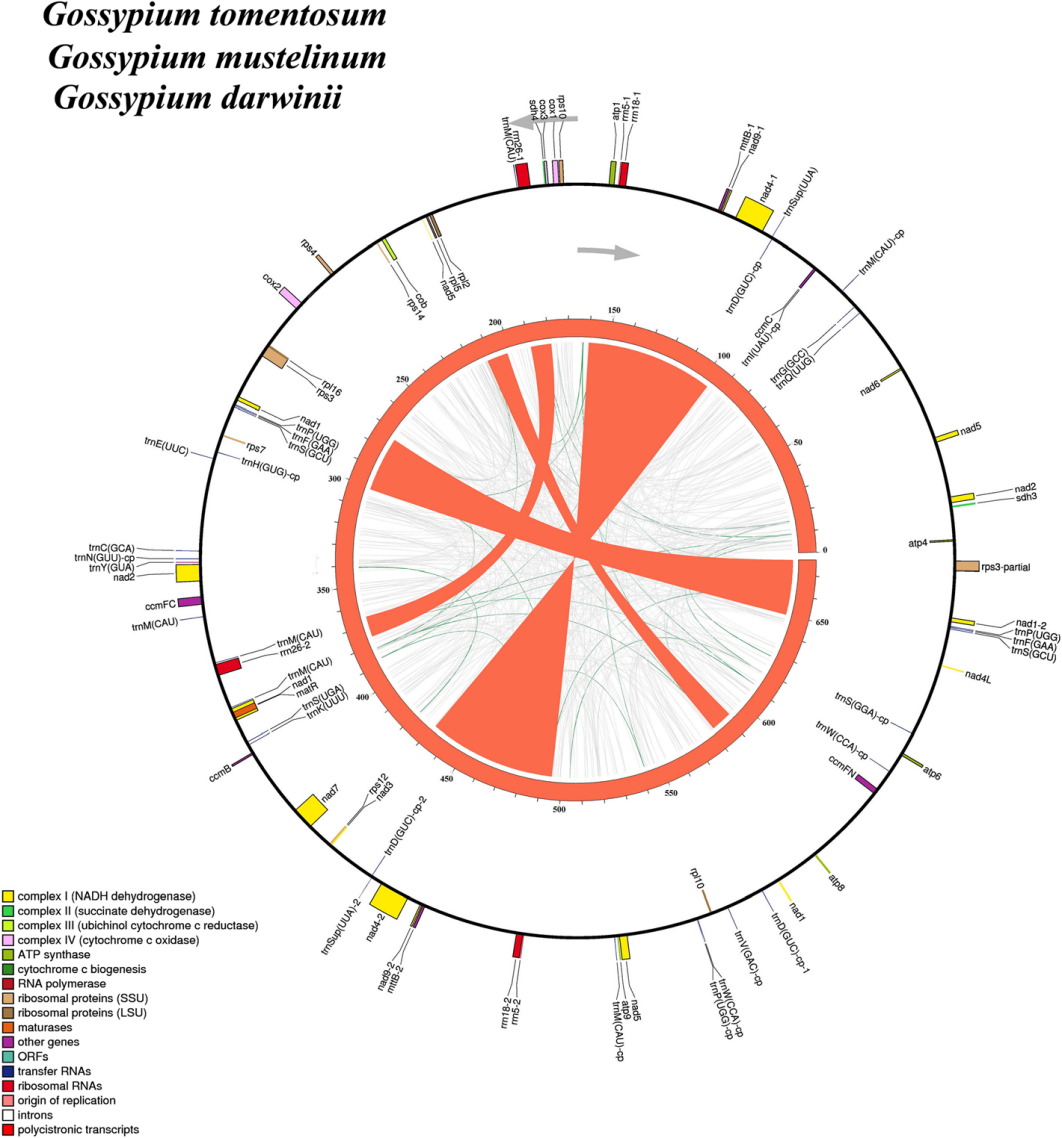
Genome maps of three allotetraploid *Gossypium* mitogenomes. The map explanations were the same to Fig. 1

Six *Gossypium* mitochondrial genomes were obtained in present study, including three diploid D species and three allotetraploid AD species. Complete mitochondrial DNA sequences were deposited in the GenBank database respectively: *G. thurberi* D_1_ (Accession No. KR736343), *G. davidsonii* D_3-d_ (Accession No. KR736344), *G. trilobum* D_8_(Accession No. KR736346), *G. tomentosum* AD_3_ (Accession No. KX388135), *G. mustelinum* AD_4_ (Accession No. KX388136) and *G. darwinii* AD_5_ (Accession No. KX388137) (Table 1). The six *Gossypium* mitogenomes were all assembled as single circular molecules of lengths about 644 kb in diploid (Additional file 1: Figure S1A) and 677 kb in allotetraploid (Additional file 1: Figure S1B), respectively. The genomic structures were identical within diploid group (Fig. 1) and allotetraploid group (Fig. 2), respectively, but differed between the two groups. The diploid group had six large repeats (>1 kb) whereas the allotetraploid group had four large repeats (Fig. 1; Fig. 2), which may be involved in the rearranged mitogenome organizations in diploid and allotetraploid *Gossypium*.

**Table 1:** Main features of the ten assembled *Gossypium* mitogenomes

Genome Characteristics	*G. thurberi*	*G. davidsonii*	*G. raimondii*	*G. trilobum*	*G. arboreum*	*G. hirsutum*	*G. barbadense*	*G. tomentosum*	*G. mustelinum*	*G. darwinii*
Accession	KR736343	KR736344	KR736345	KR736346	KR736342	JX065074	KP898249	KX388135	KX388136	KX388137
Genome groups	D_1_	D_3-d_	D_5_	D_8_	A_2_	AD_1_	AD_2_	AD_3_	AD_4_	AD_5_
Genome size (bp)	644,395	644,311	643,914	644,460	687,482	621,884	677,434	677,295	677,306	677,286
Circular chromosomes	1	1	1	1	1	1	1	1	1	1
Percent G + C content	44.92	44.94	44.94	44.92	44.94	44.95	44.98	44.98	44.98	44.97
Protein genes	36	36	36	36	39	36	39	39	39	39
tRNA genes	27	27	27	27	30	29	30	30	30	30
Native	17	17	17	17	17	17	17	17	17	17
Plastid-derived	10	10	10	10	13	12	13	13	13	13
tRNAs with introns	3	3	3	3	4	3	4	4	4	4
rRNA genes	7*	7*	7*	7*	7*	4	6	6	6	6
Large repeats: >1 kb(number)	6	6	6	6	4	4	4	4	4	4

Comparison of mitochondrial gene content among *Gossypium* species reveals a conserved pattern of evolutionary stasis for diploid and allotetraploid species, respectively (Table 1). The *Gossypium* mitogenomes contain 36 protein coding genes with five genes (*rps1*, *rps2*, *rps11*, *rps13* and *rps19*) being lost during coevolution with nucleus, compared to the common ancestor of seed plants [65, 66]. The repeat sequences confer some redundant gene copies (*nad4*, *nad9* and *mttB*) in three allotetraploid species with uncertain functions (Additional file 2: Table S1). These mitochondrial genomes (Table 1) show high identity in gene content but no similarity in genome organization (Fig. 1; Fig. 2) with each other or with previously published cotton mitochondrial genomes [37–39], with apparent major differences in genome organization and size.

### Syntenic regions and rearrangement

**Fig. 3:**
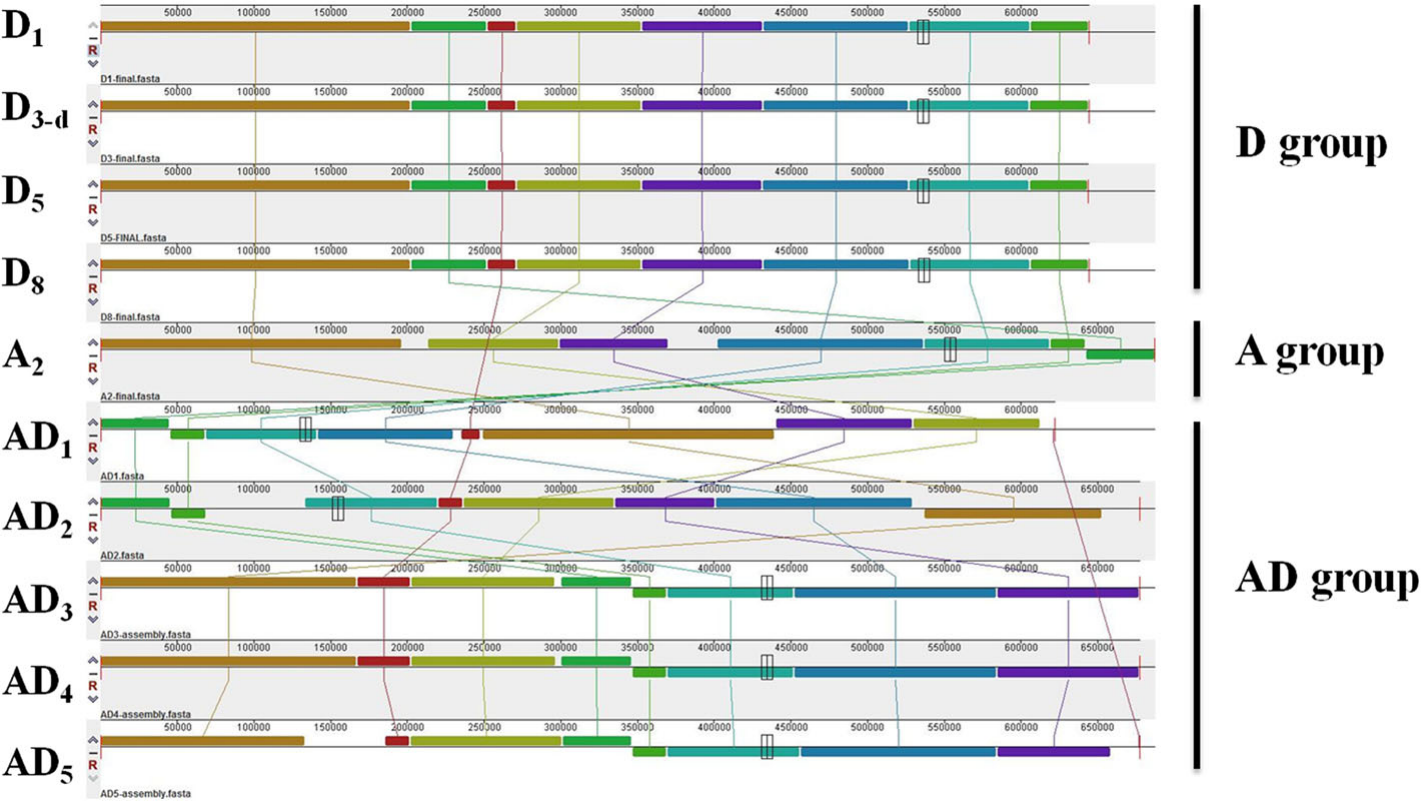
Progressive Mauve show the genome size variation and the global rearrangement structure of 10 mitochondrial chromosomes among *Gossypium*. The mitogenome of *G. arboreum* (A_2_) is the largest with a circular DNA molecule of 687,482 base pairs (bp) while the smallest mitogenome (from *G. hirsutum* AD_1_) is only 621,884 bp. Each genome is laid out horizontally and homologous segments are shown as colored blocks connected across genomes. Blocks that are shifted downward in any genome represented segments that are inverted relative to the reference genome (*G. thurberi* D_1_)

After combining four other *Gossypium* mitochondrial genomes [37–39], totally, ten species of five allotetraploid and five diploid were used for analyses: one from A genome, four from D genome and five from AD genome groups, respectively (Table 1). Syntenic regions were identified between ten *Gossypium* mitochondrial genomes with eight large major syntenic blocks (Fig. 3). The genomic structures were totally identical in four species of D group, indicating that the mitochondrial genome structures may be highly conserved in D genome species. *G. trilobum* (D_8_) contributed the cytoplasmic male sterility (CMS) cytoplasm in cotton [67–69], however, no genome rearrangement or large indel segments variations compared with mito-genomes of other D species, implying that the mitochondrial CMS-associated gene in cotton may function with different mechanism.

In addition, compared to D group species, the mitochondrial genomic structure in A group (A_2_) was highly rearranged (Fig. 3). Interestingly, genome rearrangements also occurred among the five allotetraploid species, as was already reported in the *G. hirsutum* - *G. barbadense* comparison [38]. Despite the fact that three allotetraploid species (*G. tomentosum* AD_3_, *G. mustelinum* AD_4_ and *G. darwinii* AD_5_) exhibited the same genome organization, a disorder existed between the mitogenomes of *G. hirsutum* AD_1_ and *G. barbadense* AD_2_ (Fig. 3).

### Gene order and repeat sequences

To uncover the formation mechanism of recombination generating multiple genomic arrangements in *Gossypium*, we presented the gene order with five major linear models and genes located in repeat regions shown in bold (Additional file 3: Figure S2). The gene orders in the D genome species are highly conserved but not identical to that in either *G. arboreum* (A_2_) or the AD groups with six-seven gene clusters scattered. Though there exists a few changes in mitochondrial gene order within each of five models in the three *Gossypium* lineages as shown by ten released mitogenomes, a minimum of two and three changes (inversions and translocations) need to be invoked to explain the differences of gene order among diploid and allotetraploid *Gossypium*, respectively (Fig. 3), and how these genomic rearrangements events happened are difficult to reconstruct. Repeat sequences have been suggested to serve as sites of homologous recombination, resulting in gene order changes in mitochondrial genomes [19].

The repeat sequences detected in the *Gossypium* mitogenomes in present and earlier studies [37–39] may be responsible for mitochondrial gene order changes between diploids and allotetraploids (Table 2). There mainly existed six or four large repeats in D group or (A + AD) groups, respectively. The repeat sizes were almost identical in D group but differed in (A + AD) groups. Despite a big deletion, about 50 kb that occurred in R1 of *G. hirsutum* (AD_1_), a 27 kb repeat was unique to the AD group. In addition, the repeat diverged considerately between the two diploid *Gossypium* groups (Table 2). In addition, genes in the border of the gene clusters in *Gossypium* were almost located in or close to the repeat sequences (Additional file 3: Figure S2). These variations in repeat sequences may perhaps cause the major inversions and translocations within the mitochondrial genome of the common ancestor shared by D-A and A-AD species after *Gossypium* had diverged. Evolution of gene order in diploid D group mitogenomes of *Gossypium* is overall quite conservative, but exists divergence between different diploid and allotetraploid lineages.

**Table 2:** Major repeat sizes (>1 kb) in *Gossypium* mitochondrial genomes

*Gossypium* species	Repeat 1 (kb)	Repeat 2 (kb)	Repeat 3 (kb)	Repeat 4 (kb)	Repeat 5 (kb)	Repeat 6 (kb)
*G. thurberi*	12,921	12,669	10,632	9121	8544	7317
*G. davidsonii*	12,937	12,669	10,624	9121	8544	7317
*G. raimondii*	12,741	12,670	10,624	9121	8544	7317
*G. trilobum*	12,921	12,669	10,650	9121	8544	7317
*G. arboreum*	63,789	32,975	15,029	10,246		
*G. hirsutum*	10,302	27,495	10,623	10,251		
*G. barbadense*	63,904	26,936	10,615	10,246		
*G. tomentosum*	63,888	27,425	10,614	10,246		
*G. mustelinum*	63,886	27,425	10,614	10,246		
*G. darwinii*	63,893	27,425	10,614	10,246		

### Conservation and variants in *Gossypium* mitochondrial genomes

Considering that all the *Gossypium* mitogenomes have similar genome complexity, comparative analysis were conducted to determine the proportion of the sequences that each shared in common with the others (Table 3). One of the most surprising outcomes is how rapidly sequence segments were gained or lost. Genome specific fragments is not present in any two genomes of the D or AD groups, respectively (Table 3). While reciprocity is generally not seen in any other comparisons, even between the two diploid mitogenome groups: *G. arboreum* (A_2_) lost 2.97% of the sequences present in D group, but D group lost 0.77% of the A species’ sequences. *G. arboreum* (A_2_) is attributed to be the putative maternal contributor to the progenitor of AD group [21, 34, 70], however, each of the five AD group genomes has lost substantial amounts of sequence that is present in the *G. arboreum* (A_2_) genomes and vice versa (Table 3). The difference is more striking when comparing mitogenomes of D group and AD group: D group species lost only 0.64% of the AD group mitogenomes, but AD group species lost 4.41% of the D group mito-genomes. Reciprocal differences were more apparent in the comparisons between male-fertile and CMS (cytoplasmic male sterility) mitochondrial genomes [71–73].

**Table 3:** Percentage of *Gossypium* mitochondrial genome complexity that is absent in other genomes

	vs	D_1_	D_3-d_	D_5_	D_8_	A_2_	AD_1_	AD_2_	AD_3_	AD_4_	AD_5_
References genomes	D_1_		0	0	0	0.77	0.64	0.64	0.64	0.64	0.64
	D_3-d_	0		0	0	0.77	0.64	0.64	0.64	0.64	0.64
	D_5_	0	0		0	0.77	0.64	0.64	0.64	0.64	0.64
	D_8_	0	0	0		0.77	0.64	0.64	0.64	0.64	0.64
	A_2_	2.97	2.97	2.97	2.97		0.12	0.12	0.12	0.12	0.12
	AD_1_	4.41	4.41	4.41	4.41	1.63		0	0	0	0
	AD_2_	4.41	4.41	4.41	4.41	1.63	0		0	0	0
	AD_3_	4.41	4.41	4.41	4.41	1.63	0	0		0	0
	AD_4_	4.41	4.41	4.41	4.41	1.63	0	0	0		0
	AD_5_	4.41	4.41	4.41	4.41	1.63	0	0	0	0	

Note: Small deletions (< 100 bp) are not considered to be missing segments. D_1_ = *G. thurberi*, D_3-d_ = *G. davidsonii*, D_5_ = *G. raimondii*, D_8_ = *G. trilobum*, A_2_ = *G. arboreum*, AD_1_ = *G. hirsutum*, AD_2_ = *G. barbadense*, AD_3_ = *G. tomentosum*, AD_4_ = *G. mustelinum*, AD_5_ = *G. darwinii*

**Fig. 4:**
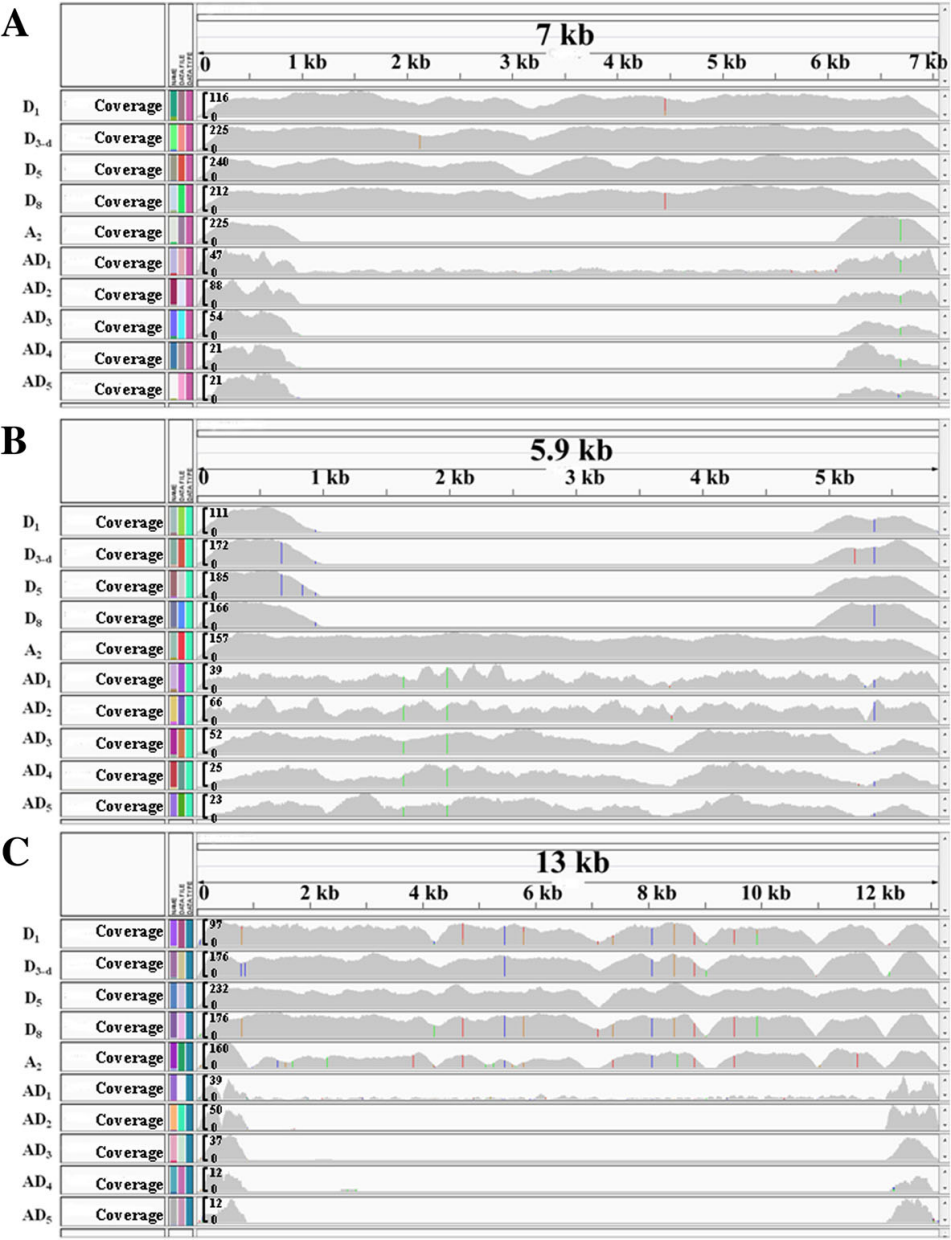
Observed coverage of mapped paired-end reads supporting the existence of a large insertion or deletion in *Gossypium* species. IGV screenshot of the variability and coverage observed in ten *Gossypium* sequence samples. Upper panel represent the unique sequences coordinates. There are ten panels corresponding to the different *Gossypium* sequences. The track in each of these panels describes the density of read mapping or coverage depth. **a**: unique segment in D group. **b**: unique segment in A + AD group. **c**: unique segment in diploid groups

In fact, the genome complexity in *Gossypium* presented eight unique segments ranging from 108 bp to 7888 bp in length in D group mitochondrial genomes, comprising a total of 18,194 bp (Indels of <100 bp were not included) (Fig. 4a, showing one of the unique segments); while three specific fragments detected in (A + AD) group mitochondrial genomes with the largest size 3876 bp in length, 4315 bp in total (Fig. 4b). In addition, a large segment more than 11 kb in length that is present in the diploid mitogenome is not present in any of the other five allotetraploid mitogenomes (Fig. 4c). Despite the fact that the ancestor of A-genome group is the maternal source of extant allotetraploid species [20–23, 34], unique presence/absence variations existed as well (Fig. 4c; Additional file 4: Figure S3).

### RNA editing in *Gossypium* mitochondrial genes

Post-transcriptional RNA-editing of mitochondrial genes is both ubiquitous and important for regulation [74]. Typically, RNA editing of mitochondrial transcripts in flowering plants occurs in coding regions of mitochondrial transcripts to convert specific cytosine residues to uracil (C → U) [75, 76]. For ten *Gossypium* species, we predicted sites of C-to-U editing using the PREP-Mt. online tool [59] with a cutoff of 0.2. The number of predicted C-to-U edits across the entire coding regions of their shared 36 protein genes is almost similar for ten *Gossypium* species (451), with one editing site lower in *G. hirsutum* (450 sites) caused in *nad3* gene (Table 4). The simplest interpretation of these results is that the whole of edit sites in ten *Gossypium* species were present in their common ancestor, while the species-specific sites are less derived in cotton.

**Table 4:** The numbers of edit sites in *Gossypium* mitochondrial protein-coding genes

Genes	*G. thurberi*	*G. davidsonii*	*G. raimondii*	*G. trilobum*	*G. arboreum*	*G. hirsutum*	*G. barbadense*	*G. tomentosum*	*G. mustelinum*	*G. darwinii*
*atp1*	6	6	6	6	6	6	6	6	6	6
*atp4*	11	11	11	11	11	11	11	11	11	11
*atp6*	9	9	9	9	9	9	9	9	9	9
*atp8*	3	3	3	3	3	3	3	3	3	3
*atp9*	6	6	6	6	6	6	6	6	6	6
*ccmB*	29	29	29	29	29	29	29	29	29	29
*ccmC*	24	24	24	24	24	24	24	24	24	24
*ccmFc*	13	13	13	13	13	13	13	13	13	13
*ccmFn*	31	31	31	31	31	31	31	31	31	31
*cob*	9	9	9	9	9	9	9	9	9	9
*cox1*	16	16	16	16	16	16	16	16	16	16
*cox2*	12	12	12	12	12	12	12	12	12	12
*cox3*	5	5	5	5	5	5	5	5	5	5
*matR*	15	15	15	15	15	15	15	15	15	15
*mttB*	25	25	25	25	25	25	25	25	25	25
*nad1*	19	19	19	19	19	19	19	19	19	19
*nad2*	25	25	25	25	25	25	25	25	25	25
*nad3*	13	13	13	13	13	12	13	13	13	13
*nad4*	29	29	29	29	29	29	29	29	29	29
*nad4L*	11	11	11	11	11	11	11	11	11	11
*nad5*	24	24	24	24	24	24	24	24	24	24
*nad6*	12	12	12	12	12	12	12	12	12	12
*nad7*	23	23	23	23	23	23	23	23	23	23
*nad9*	8	8	8	8	8	8	8	8	8	8
*rpl2*	4	4	4	4	4	4	4	4	4	4
*rpl5*	10	10	10	10	10	10	10	10	10	10
*rpl10*	4	4	4	4	4	4	4	4	4	4
*rpl16*	6	6	6	6	6	6	6	6	6	6
*rps3*	10	10	10	10	10	10	10	10	10	10
*rps4*	17	17	17	17	17	17	17	17	17	17
*rps7*	2	2	2	2	2	2	2	2	2	2
*rps10*	5	5	5	5	5	5	5	5	5	5
*rps12*	4	4	4	4	4	4	4	4	4	4
*rps14*	3	3	3	3	3	3	3	3	3	3
*sdh3*	4	4	4	4	4	4	4	4	4	4
*sdh4*	4	4	4	4	4	4	4	4	4	4
*Total*	451	451	451	451	451	450	451	451	451	451
*Edits/gene*	12.5	12.5	12.5	12.5	12.5	12.5	12.5	12.5	12.5	12.5

### Mitochondrial genome evolution in *Gossypium*

**Fig. 5:**
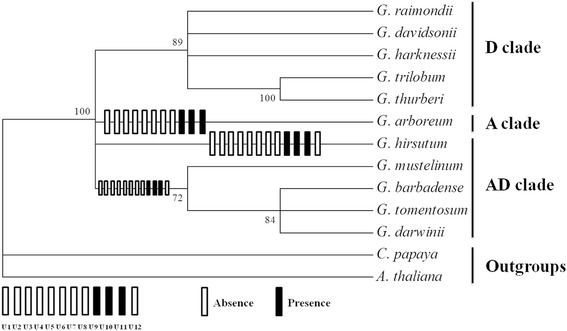
Maximum likelihood (ML) phylogenetic tree of ten *Gossypium* species was constructed based on nucleotide sequences of 36 mitochondrial genes. Bootstrap values for all major divergences were high (>70%) on the corresponding nodes. The hollow or black bars represent unique present or absent segments in ten *Gossypium* mitogenomes

Phylogenetic relationships among 10 *Gossypium* species with two outgroups, was generated using a concatenated analysis of 36 mitochondrial protein-coding genes (Fig. 5). The topology of the resulting tree supports *G. arboreum* as the maternal donor to polyploid cotton species, which further supports our former result [39]. We mapped these specific indels into the phylogenetic clades, as shown in Fig. 5, which implied an ongoing dynamic divergence process. First, eight mitogenome fragments (U1-U8) were involved in loss events after *G. arboreum* (A_2_) diverged from a common ancestor shared with the D genome group. Subsequently, three genome fragments (U9-U11) were transferred from the nucleus to the mitochondrial genome in an A-genome ancestor or contributor/donator before the formation of the allopolyploidization event. Finally, a large genome fragment (U12), about 11 kb, was lost during the divergence process of allotetraploid ancestor species (Fig. 5). This 11 kb deletion (corresponding to U12 in diploid mitogenomes) was adjacent to the specific repeat sequence R2 in AD group, which might lead to the formation of R2 repeat sequences (unique to AD group species) during evolution. In addition, variations in repeat and Indels lengths also cause the great difference of *Gossypium* mitochondrial genome sizes (Fig. 3 and Fig. 5). MtDNA intergenic regions are known to possess more unique segments than genic regions, however, shorter repeats account for the relatively small size of the D-group mitochondrial genomes. Interestingly, a large deletion ~50 kb in length in R1 may lead to the small size of *G. hirsutum* (AD_1_) [37], compared to the other four allotetraploid genomes. Comparative mitochondrial genome analysis revealed rapid mitochondrial genome rearrangement and evolution even within a single subspecies.

**Fig. 6:**
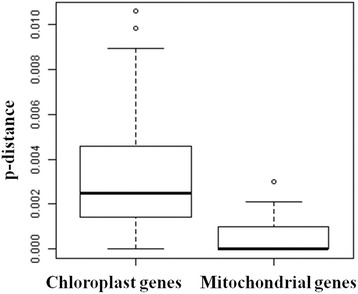
Distribution of p-distances from 78 chloroplast and 36 mitochondrial protein-coding exons among 10 *Gossypium* species

In addition, we calculated the p-distances representing evolutionary divergence from 78 chloroplast and 36 mitochondrial protein-coding exons among 10 *Gossypium* species, as shown in Fig. 6. Here, the average evolutionary divergence was 0.0031 in chloroplast genes but only 0.0005 in mitochondrial genes among 10 *Gossypium* species. The mitochondrial genes were highly conserved with low evolutionary divergence, however, their genome structures displayed the extremely rapid evolution of various changes, including repeat and large indels variations. Based on these results, the evolutionary distance of mitochondrial genomes are much lower than the chloroplast genomes in *Gossypium*, however, rapid varying mitogenome structures evolves much faster than the highly conserved chloroplast genomes [35–39].

### MtDNAs insert into the nuclear chromosomes in *Gossypium*

**Fig. 7:**
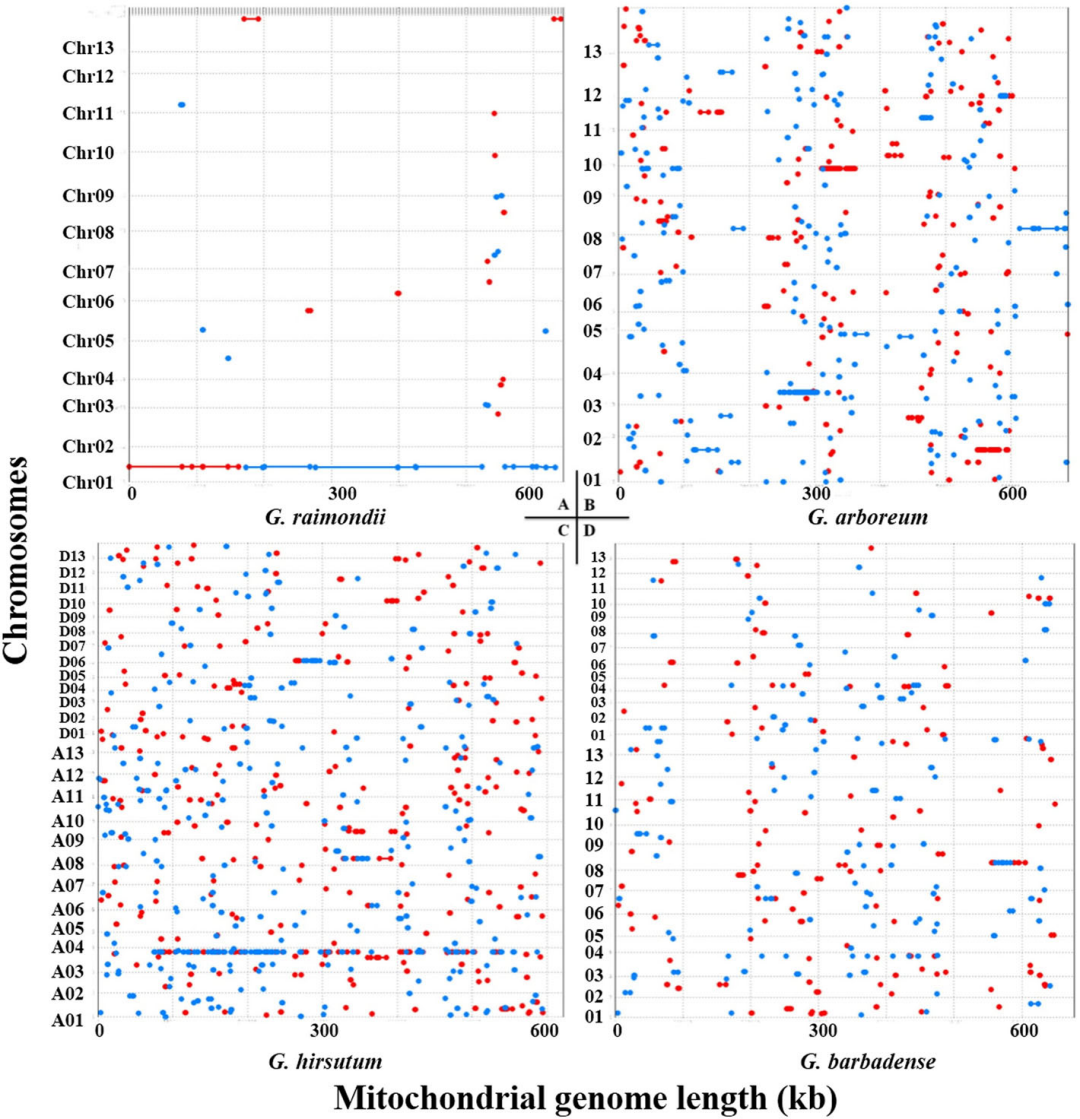
Mitochondrial DNAs insertions into four *Gossypium* nuclear genomes detected by whole-genome alignment. The results were filtered to select only those alignments which comprise the one-to-one mapping between reference and query, and then display a dotplot of the selected alignments. The red and blue lines refer positive and reverse matches, respectively. **a**: Dot matrix analysis of *numts* in *Gossypium raimondii* (D_5_) nuclear genome performed using MUMmer (Delcher et al., 2002). **b**: Dot matrix analysis of *numts* in *G. arboreum* (A_2_) nuclear genome. **c**: Dot matrix analysis of *numts* in *G. hirsutum* (AD_1_) nuclear genome. **d**: Dot matrix analysis of *numts* in *G. barbadense* (AD_2_) nuclear genome

In this study, four sets of mitochondrial and nuclear genomes of *Gossypium* species (two diploids and two allotetraploid) were analyzed. Numts in four *Gossypium* nuclear genomes were detected by whole-genome alignment. Dot matrix analysis of mitochondrial vs nuclear genomes in *G. raimondii* (D_5_) show that there is a stretch of ~598 kb (92.91%) of sequence that is nearly identical to that of the *G. raimondii* mitochondrial genome (Fig. 7a) in chromosome 1. This insertion is at least 99.80% identical to the mitochondrial genome, suggesting that the transfer event was very recent. The organization of the assembled mitochondrial genome differs from that of the mitochondrial DNA in the nucleus with an internal deletion (Fig. 7a), which might occur during or after transferring and represent an alternate isoform of the *G. raimondii* mitochondrial genome. In addition, *G. hirsutum* also has a nearly complete NUMT on chromosome A03 (Fig. 7c), and small to median-large fragments of mitochondrial DNA have been identified in three *Gossypium* species nuclear genomes (Fig. 7b-d), showing apparently sporadic fragmentation compared to *G. raimondii*. So much noise in *Gossypium* nuclear chromosomes of (Fig. 7b-d) are just repetitive derived elements. These results may be caused by the insertion of retrotransposon elements into mitochondrial DNA insertions that may contribute significantly to their fragmentation process in the other three nuclear genomes.

In addition, most *numts* had >99% nucleotide identity to the homologous organelle sequences, so the lack of divergence in *G. raimondii* indicates that they must have been transferred to the nucleus recently. In order to estimate how old these insertions are, p-distance rates and some estimates of rate/million years between mitochondrial sequences and numts were studied here. We have dated 20 larger NUMTs in *G. raimondii* (Additional file 5: Table S2), 16 larger NUMTs in *G. arboreum* (Additional file 6: Table S3), 15 larger NUMTs in *G. hirsutum* (Additional file 7: Table S4) and 12 larger NUMTs in *G. barbadense* (Additional file 8: Table S5). These data showed that the insertion time of NUMTs was close among one chromosome, but with big divergence between different chromosomes. For example, the different insertion time for five larger NUMTs in chromosome A03 of *G. hirsutum* (ranging from 0.33–1.03 MYA), with other chromosomes (insertion time ranging from 0.91–11.43 MYA) (Additional file 7: Table S4).

**Fig. 8:**
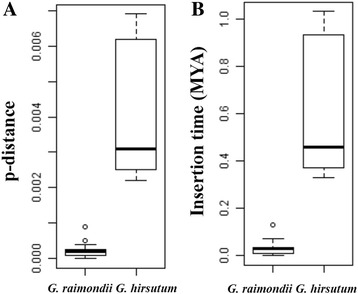
p-distances (**a**) and estimated divergence time (**b**) of two recent nearly full length insertion in *G. raimondii* (Chr01, Fig. 7a) and *G. hirsutum* (Chr A03, Fig. 7c)

The p-distance of larger NUMTs ranged from 0~0.0009 in chromosome 01 of *G. raimondii* (Fig. 8a; Additional file 5: Table S2) and 0.0022~0.0069 in chromosome A03 of *G. hirsutum* (Fig. 8a; Additional file 7: Table S4). We have dated these larger NUMTs in chromosome 01 of *G. raimondii* with insertion time ranging from 0~0.13 MYA (Fig. 8b; Additional file 5: Table S2), and from 0.33~1.03 MYA in chromosome A03 of *G. hirsutum* (Fig. 8b; Additional file 7: Table S4). These results revealed that two nearly full length insertion events in *G. raimondii* (Chr01, Fig. 7a) and *G. hirsutum* (chromosome A03, Fig. 7c) occurred recently.

## Discussion

From the perspective of divergence, *Gossypium* originated from a common ancestor approximately ten million years ago and an allopolyploidization event occurred approximately 1.5 million years ago [35, 36]. Plant mitochondrial genomes have experienced myriad synteny-disrupting rearrangements even over a very short evolutionary timescale. Like most angiosperm mitogenomes abundant in repeat sequences with larger repeats mediating recombination at moderate to high frequency [19, 77], these recombination events generated multiple mito-genomic arrangements differed in *Gossypium* genome groups, which may be largely caused by both larger repeats and some key InDels or SVs during evolution, and quickly eroded synteny even among closely related plants [8, 72, 73, 78].

These cotton mitochondrial genomes diverged much, as indicated by the InDels events unique to A genome species, D and AD groups, respectively. All these structural variants (SVs) are located in intergenic regions of mitogenomes. Some of them overlapped with their breakpoints and junctions occurring in repetitive and homologous genomic regions. And insertions or deletions were mostly generated from crossovers of repetitive regions or homologous regions [79].

There existed apparent inversions and translocations, which can offer clues to explain gene order differences of mitogenomes between different *Gossypium* groups. For examples, the mode of gene order changed by inversions and/or translocations was presented in early land plant mitochondrial genomes evolution of bryophytes [80] as well as rapidly rearranged mitochondrial genomes of vascular plants [71–73, 81, 82]. Apart from apparent rearranged mitogenome organizations in diploid and allotetraploid *Gossypium*, mitochondrial genome rearrangements have also been detected in diploid and allotetraploid species of *Brassica* [74, 83]. Generally, apparent variations in the mitogenome structures were always tested to be associated with cytoplasmic male sterility (CMS) and its maintainer lines [71–73], thus a new mitochondrial gene was produced by recombination and conferred CMS with its encoded protein interacted with the nuclear encoded mitochondrial protein to cause a detrimental interaction [43]. However, no genome rearrangement or large indel segments variations compared with mito-genomes of other D species, implying that the mitochondrial CMS-associated gene in cotton may function with different mechanism.

In addition, RNA-editing sites in *Gossypium* may not be in charge of cytoplasmic male sterility in D_8_ cotton. RNA editing events have been compared in eight mitochondrial genes (*atp1*, *atp4*, *atp6*, *atp8*, *atp9*, and *cox1*, *cox2*, *cox3*) among CMS-D_8_ three lines in cotton [75]. Although the frequencies of RNA editing events between mtDNA genes were different, no differences between cotton cytoplasms that could account for the CMS phenotype or restoration. In view of these results, the complete mitogenome sequences will provide the useful data resources for targeting the CMS-related genes in *G. trilobum* D_8_ cotton in further studies.

As for MtDNAs insert into the nuclear chromosomes in *Gossypium*, Lin et al., (1999) and Stupar et al., (2001) also identified an intact mtDNA copy on chromosome 2 in the nucleus of Arabidopsis with more than 99% identity, which proved this type of mitochondrion-to-nucleus migration event [84, 85]. Second, these mitochondrion-to-nucleus migrations proved to be the independent events after the divergence of the *Gossypium* progenitors. These genome changes within the diploid and allotetraploid *Gossypium* species is worthy of more attention in future studies.

## Conclusions

Plants mitochondrial genomes are evolutionarily intriguing because of the highly conserved genic content and slow rates of genic sequence evolution [18, 82]. These features contrasted sharply with the highly labile genomic structure, genome size, DNA repair mechanism and recombination induced by different types and origins of repeated sequences [82, 86–88]. Whole mitogenome sequences have been released in an ongoing process [9, 11, 38, 81, 89], which provide information for dissecting the evolutionary modifications in these genomes, such as gene loss [88], sequence acquisitions or loss [9], multiple sequence rearrangements [73] and dynamic structure evolution [38, 39]. Here, we presented six more cotton mitochondrial genomes, which showed apparently distinct divergence. Despite the short divergence time separating diploid and allotetraploid cotton species [35, 36], many of the hallmark features of mitochondrial genome evolution are evident, including differential genic content, genome rearrangements, inversion and translocation, gains/losses of multiple small and large repeats, presence/absence variations, and the mitogenome of *G. trilobum* D_8_ cotton for targeting CMS-associated gene. Comparative analyses illustrated that four of the outcomes are quite surprising, including: 1) how rapidly mitochondrial genome rearrangements occur within a single subspecies (diverged ~ 10 mya), 2) how rapidly mitochondrial sequence segments are gained or lost, 3) RNA editing events were almost conserved in ten *Gossypium* mitogenomes, and 4) a previous unusual report of the integration of 93% of the mitochondrial genome of *G. raimondii* into chromosome 1 is confirmed with an estimation of insertion time 0.05 MYA. Increasing insight into the mechanisms and functional consequences of plant mitochondrial genome variation are expected to be helpful to elucidate the process of rapid evolutionary divergence mechanism between closely related mitochondrial genomes.

## Additional files

Additional file 1: Figure S1. Genome maps of six diploid and allotetraploid *Gossypium* mitogenomes. Genes exhibited on the inside of outer circles are transcribed in a clockwise direction, while genes on the outside of outer circles are transcribed in a reverse direction. (JPEG 424 kb)

Additional file 2: Table S1. Gene contents of the six *Gossypium* mitogenomes. Note: Genes presented in multiple copies are denoted with a number (e.g., 2 or 3). (DOCX 18 kb)

Additional file 3: Figure S2. Gene order comparison among mitogenomes of *Gossypium*. Colored blocks represent regions of conserved gene clusters in the *Gossypium* genomes and genes in bold are located in the repeat regions. *Rpl2* and *atp8* are shown in red bold to indicate that they are just close to or partially overlapped with the repeat sequences. (JPEG 1006 kb)

Additional file 4: Figure S3. Observed coverage of mapped paired-end reads supporting the existence of a small deletion (~500 bp) in *G. arboreum* compared to AD group species. IGV screenshot of the variability and coverage observed in ten samples of *Gossypium* sequence. Upper panel represent the unique sequences coordinates. There are five panels corresponding to the different *Gossypium* sequences. The track in each of these panels describes the density of read mapping or coverage depth. (JPEG 353 kb)

Additional file 5: Table S2. Nucleotide distances and divergence time (MYA) between mitochondrial sequences and corresponding *numts* in *G. raimondii*. Note: ^a^ twenty *numts* represent the largest mitochondrial fragments transferred into the nuclear chromosomes in *G. raimondii*. (DOCX 17 kb)

Additional file 6: Table S3. Nucleotide distances and divergence time (MYA) between mitochondrial sequences and corresponding *numts* in *G. arboreum*. (DOCX 17 kb)

Additional file 7: Table S4. Nucleotide distances and divergence time (MYA) between mitochondrial sequences and corresponding *numts* in *G. hirsutum*. Note: ^a^ fifteen *numts* represent the largest mitochondrial fragments transferred into the nuclear chromosomes in *G. hirsutum*. ^b^ represents five fragments from nearly full length mitochondrial fragments transferred into the nuclear A03 chromosomes in *G. hirsutum* (Fig. 7C). (DOCX 17 kb)

Additional file 8: Table S5. Nucleotide distances and divergence time (MYA) between mitochondrial sequences and corresponding *numts* in *G. barbadense*. Note: ^a^ twelve *numts* represent the largest mitochondrial fragments transferred into the nuclear chromosomes in *G. barbadense*. (DOCX 16 kb)

## Abbreviations

CMS: Cytoplasmic male sterility
G.: *Gossypium*
IGV: Integrative Genomics Viewer
Indels: Insertions and deletions
Mitogenome: Mitochondrial genome
mtDNA: Mitochondrial DNA
ORFs: Open reading frames
rRNAs: Ribosomal RNAs
tRNAs: Transfer RNAs
