# Supplementary figures and images for: Rapid evolutionary divergence of diploid and allotetraploid Gossypium mitochondrial genomes
Source: BMC Genomics. 2017 Nov 13;18:876. doi: 10.1186/s12864-017-4282-5 (PMC5683544; doi:10.1186/s12864-017-4282-5)

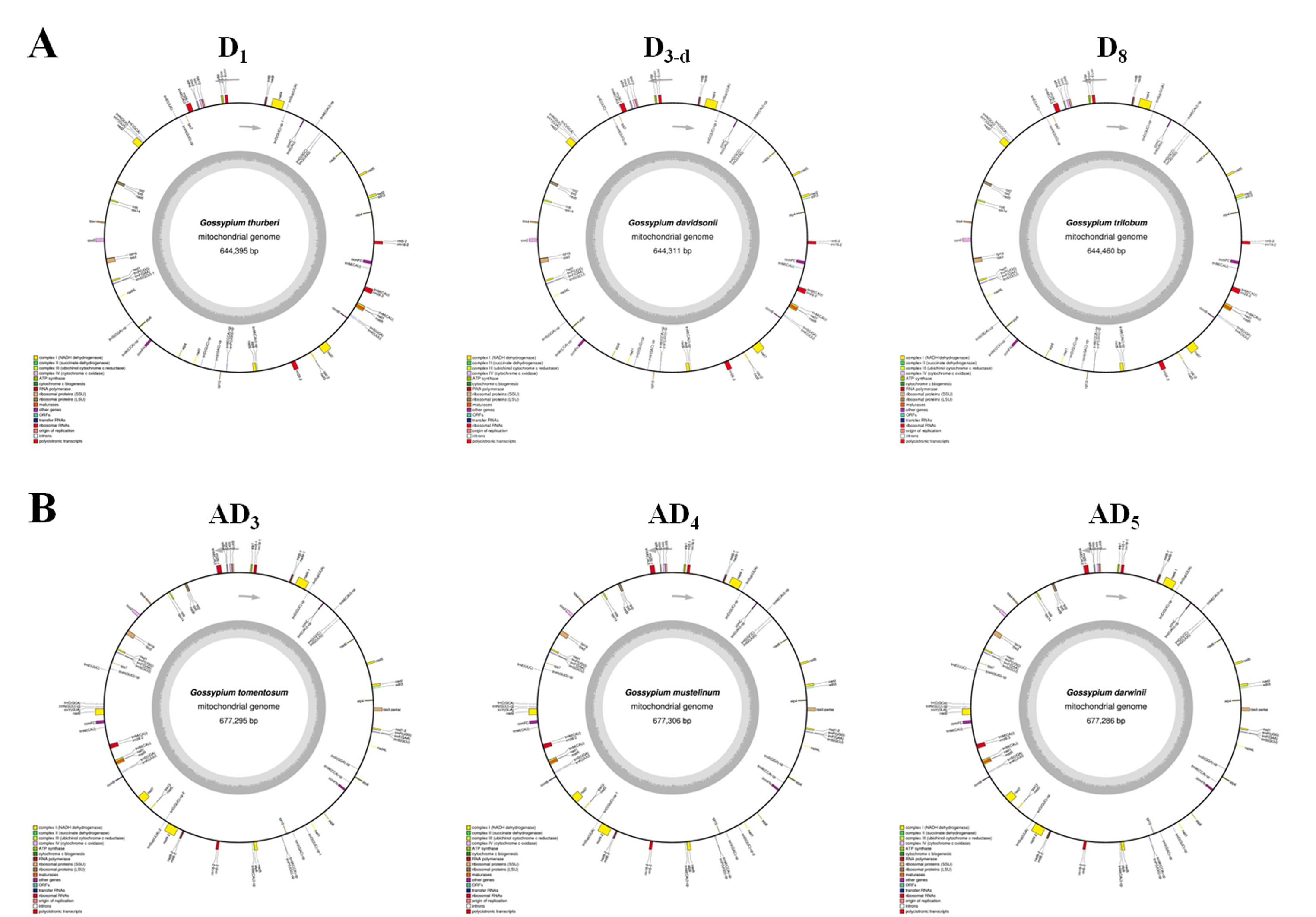

Supplement: Supplementary file 1 — Genome maps of six diploid and allotetraploid Gossypium mitogenomes. Genes exhibited on the inside of outer circles are transcribed in a clockwise direction, while genes on the outside of outer circles are transcribed in a reverse direction. (JPEG 424 kb) [file 12864_2017_4282_MOESM1_ESM.jpg]

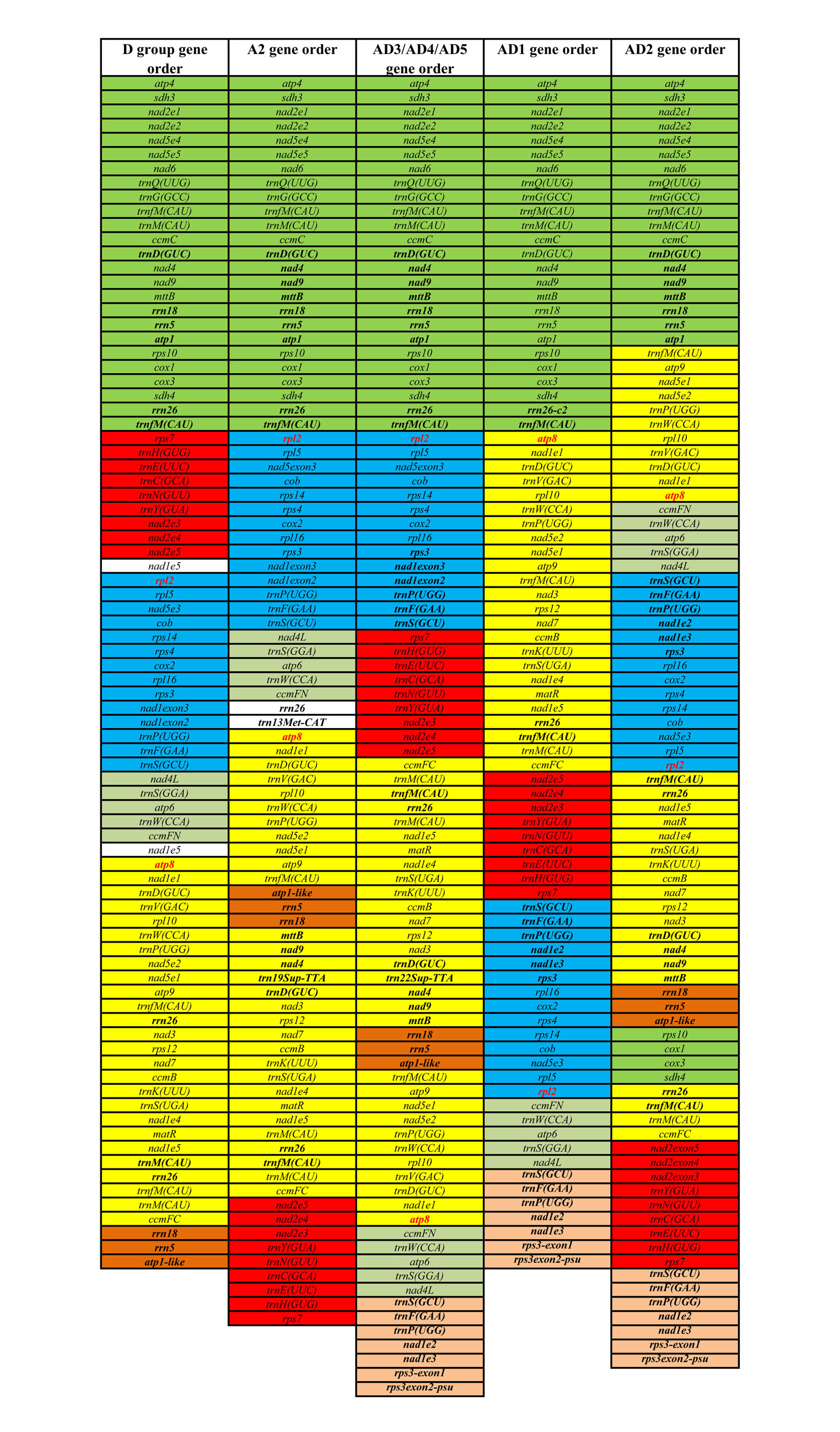

Supplement: Supplementary file 3 — Gene order comparison among mitogenomes of Gossypium. Colored blocks represent regions of conserved gene clusters in the Gossypium genomes and genes in bold are located in the repeat regions. Rpl2 and atp8 are shown in red bold to indicate that they are just close to or partially overlapped with the repeat sequences. (JPEG 1006 kb) [file 12864_2017_4282_MOESM3_ESM.jpg]

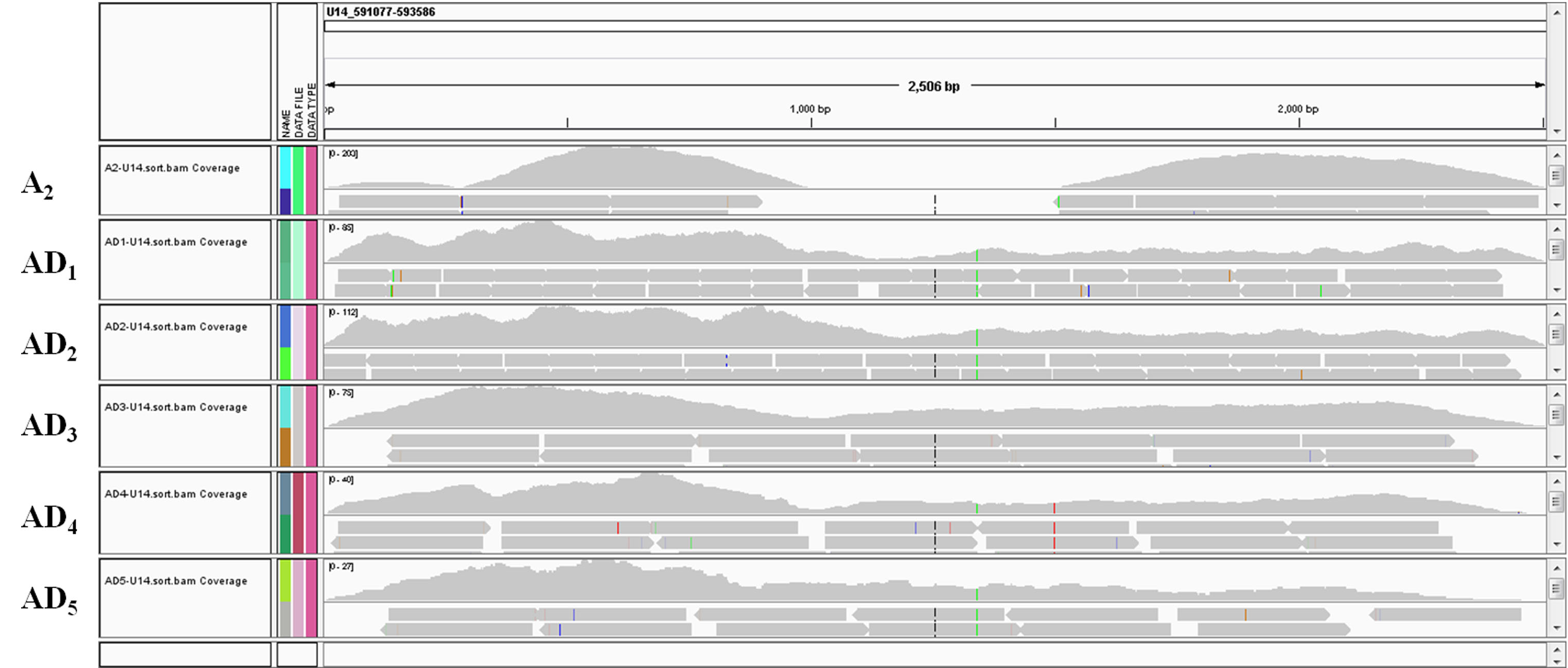

Supplement: Supplementary file 4 — Observed coverage of mapped paired-end reads supporting the existence of a small deletion (~500 bp) in G. arboreum compared to AD group species. IGV screenshot of the variability and coverage observed in ten samples of Gossypium sequence. Upper panel represent the unique sequences coordinates. There are five panels corresponding to the different Gossypium sequences. The track in each of these panels describes the density of read mapping or coverage depth. (JPEG 353 kb) [file 12864_2017_4282_MOESM4_ESM.jpg]
