# Supplementary material for: Rapid evolutionary divergence of diploid and allotetraploid Gossypium mitochondrial genomes
Source: BMC Genomics. 2017 Nov 13;18:876. doi: 10.1186/s12864-017-4282-5 (PMC5683544; doi:10.1186/s12864-017-4282-5)
Supplement: Supplementary file 2 — Gene contents of the six Gossypium mitogenomes. Note: Genes presented in multiple copies are denoted with a number (e.g., 2 or 3). (DOCX 18 kb) [file 12864_2017_4282_MOESM2_ESM.docx]

Table S1. Gene contents of the six *Gossypium* mitogenomes.

| Product group | Gene | D group | AD group | Product group | Gene | D group | AD group |
| --- | --- | --- | --- | --- | --- | --- | --- |
| complex I | *nad1* | + | + | Ribosome | *rps3* | + | + |
|  | *nad2* | + | + |  | *rps4* | + | + |
|  | *nad3* | + | + |  | *rps7* | + | + |
|  | *nad4* | + | 2 |  | *rps10* | + | + |
|  | *nad4L* | + | + |  | *rps12* | + | + |
|  | *nad5* | + | + |  | *rps14* | + | + |
|  | *nad6* | + | + |  | *rpl2* | + | + |
|  | *nad7* | + | + |  | *rpl5* | + | + |
|  | *nad9* | + | 2 |  | *rpl10* | + | + |
| complex II | *sdh3* | + | + |  | *rpl16* | + | + |
|  | *sdh4* | + | + | tRNA | *trnC(GCA)-cp* | + | + |
| complex III | *cob* | + | + |  | *trnD(GUC)-cp* | 2 | 3 |
| complex IV | *cox1* | + | + |  | *trnE(UUC)* | + | + |
|  | *cox2* | + | + |  | *trnF(GAA)* | + | + |
|  | *cox3* | + | + |  | *trnfM(CAU)-cp* | 4 | 5 |
| complex V | *atp1* | + | + |  | *trnG(GCC)* | + | + |
|  | *atp4* | + | + |  | *trnH(GUG)-cp* | + | + |
|  | *atp6* | + | + |  | *trnK(UUU)* | + | + |
|  | *atp8* | + | + |  | *trnM(CAU)* | 2 | 2 |
|  | *atp9* | + | + |  | *trnI(UAU)* | + | + |
| Cytochrome C | *ccmB* | + | + |  | *trnN(GUU)-cp* | + | + |
|  | *ccmC* | + | + |  | *trnP(UGG)* | 2 | 2 |
|  | *ccmFN* | + | + |  | *trnQ(UUG)* | + | + |
|  | *ccmFC* | + | + |  | *trnS(GCU)* | + | + |
| Other gene | *mttB* | + | 2 |  | *trnS(GGA)-cp* | + | + |
|  | *matR* | + | + |  | *trnS(UGA)* | + | + |
| rRNA | *rrn5* | 2 | 2 |  | *trnSup(UUA)* | + | 2 |
|  | *rrn18* | 2 | 2 |  | *trnV(GAC)* | + | + |
|  | *rrn26* | 3 | 2 |  | *trnW(CCA)-cp* | 2 | 2 |
|  |  |  |  |  | *trnY(GUA)* | + | + |

Genes present in multiple copies are denoted with a number (e.g., 2 or 3).
