# Supplementary material for: Rapid evolutionary divergence of diploid and allotetraploid Gossypium mitochondrial genomes
Source: BMC Genomics. 2017 Nov 13;18:876. doi: 10.1186/s12864-017-4282-5 (PMC5683544; doi:10.1186/s12864-017-4282-5)
Supplement: Supplementary file 5 — Nucleotide distances and divergence time (MYA) between mitochondrial sequences and corresponding numts in G. raimondii. Note: a twenty numts represent the largest mitochondrial fragments transferred into the nuclear chromosomes in G. raimondii. (DOCX 17 kb) [file 12864_2017_4282_MOESM5_ESM.docx]

Table S2. Nucleotide distances and divergence time (MYA) between mitochondrial sequences and corresponding *numts* in *G. raimondii*.

| Larger *NUMT*s^a^ | Length (bp) in Mitogenome | Length (bp) in Chromosome | Distribution in Chromosome | p-distance±SE | Divergence time (MYA) |
| --- | --- | --- | --- | --- | --- |
| D_5_-*Numt1* | 122,281 | 122,120 | Chr01 | 0.0002±0.0001 | 0.03±0.01 |
| D_5_-*Numt2* | 98,249 | 98,164 | Chr01 | 0.0002±0.0001 | 0.03±0.01 |
| D_5_-*Numt3* | 78,104 | 78,104 | Chr01 | 0.0000±0.0000 | 0.00±0.00 |
| D_5_-*Numt4* | 67,381 | 67,299 | Chr01 | 0.0002±0.0001 | 0.03±0.01 |
| D_5_-*Numt5* | 37,162 | 37,198 | Chr01 | 0.0000±0.0000 | 0.00±0.00 |
| D_5_-*Numt6* | 27,144 | 27,140 | Chr01 | 0.0002±0.0001 | 0.03±0.01 |
| D_5_-*Numt7* | 26,499 | 26,478 | Chr01 | 0.0003±0.0001 | 0.04±0.01 |
| D_5_-*Numt8* | 24,301 | 24,292 | Chr01 | 0.0001±0.0001 | 0.01±0.01 |
| D_5_-*Numt9* | 16,366 | 16,361 | Chr01 | 0.0001±0.0001 | 0.01±0.01 |
| D_5_-*Numt10* | 15,378 | 15,375 | Chr01 | 0.0002±0.0001 | 0.03±0.01 |
| D_5_-*Numt11* | 14,298 | 14,281 | Chr01 | 0.0004±0.0002 | 0.06±0.03 |
| D_5_-*Numt12* | 14,033 | 14,033 | Chr01 | 0.0001±0.0001 | 0.01±0.01 |
| D_5_-*Numt13* | 13,750 | 13,745 | Chr01 | 0.0005±0.0002 | 0.07±0.03 |
| D_5_-*Numt14* | 12,840 | 12,830 | Chr01 | 0.0009±0.0002 | 0.13±0.03 |
| D_5_-*Numt15* | 12,670 | 12,670 | Chr01 | 0.0000±0.0000 | 0.00±0.00 |
| D_5_-*Numt16* | 20,626 | 20,539 | Chr13 | 0.0174±0.0011 | 2.60±0.16 |
| D_5_-*Numt17* | 10,038 | 10,016 | Chr13 | 0.0082±0.0009 | 1.22±0.13 |
| D_5_-*Numt18* | 9,304 | 9,290 | Chr13 | 0.0203±0.0016 | 3.03±0.24 |
| D_5_-*Numt19* | 6,686 | 6,639 | Chr13 | 0.0128±0.0015 | 1.91±0.22 |
| D_5_-*Numt20* | 6,023 | 6,007 | Chr13 | 0.0216±0.0017 | 3.22±0.25 |

Note: ^a^ twenty *numts* represent the largest mitochondrial fragments transferred into the nuclear chromosomes in *G. raimondii*.
