# Supplementary material for: Rapid evolutionary divergence of diploid and allotetraploid Gossypium mitochondrial genomes
Source: BMC Genomics. 2017 Nov 13;18:876. doi: 10.1186/s12864-017-4282-5 (PMC5683544; doi:10.1186/s12864-017-4282-5)
Supplement: Supplementary file 6 — Nucleotide distances and divergence time (MYA) between mitochondrial sequences and corresponding numts in G. arboreum. (DOCX 17 kb) [file 12864_2017_4282_MOESM6_ESM.docx]

Table S3. Nucleotide distances and divergence time (MYA) between mitochondrial sequences and corresponding *numts* in *G. arboreum*.

| Larger *NUMT*s^a^ | Length (bp) in Mitogenome | Length (bp) in Chromosome | Distribution in Chromosome | p-distance±SE | Divergence time (MYA) |
| --- | --- | --- | --- | --- | --- |
| A_2_-*Numt1* | 20,033 | 20,033 | chr1 | 0.0007±0.0002 | 0.10±0.03 |
| A_2_-*Numt2* | 10,664 | 10,664 | chr1 | 0.0002±0.0001 | 0.03±0.01 |
| A_2_-*Numt3* | 13,243 | 13,243 | chr1 | 0.0014±0.0003 | 0.21±0.04 |
| A_2_-*Numt4* | 14,141 | 14,141 | chr2 | 0.0018±0.0004 | 0.27±0.06 |
| A_2_-*Numt5* | 17,706 | 17,706 | chr4 | 0.0008±0.0002 | 0.12±0.03 |
| A_2_-*Numt6* | 18,064 | 18,063 | chr4 | 0.0001±0.0001 | 0.01±0.01 |
| A_2_-*Numt7* | 12,478 | 12,317 | chr7 | 0.0267±0.0015 | 3.99±0.22 |
| A_2_-*Numt8* | 32,870 | 32,852 | chr8 | 0.0114±0.0004 | 1.70±0.06 |
| A_2_-*Numt9* | 20,695 | 20,646 | chr8 | 0.0102±0.0007 | 1.52±0.10 |
| A_2_-*Numt10* | 14,711 | 14,691 | chr8 | 0.0082±0.0007 | 1.22±0.10 |
| A_2_-*Numt11* | 12,113 | 12,101 | chr8 | 0.0093±0.0008 | 1.39±0.12 |
| A_2_-*Numt12* | 12,351 | 12,351 | chr11 | 0.0009±0.0002 | 0.13±0.03 |
| A_2_-*Numt13* | 14,561 | 14,561 | chr12 | 0.0018±0.0004 | 0.27±0.06 |
| A_2_-*Numt14* | 13,855 | 13,855 | chr12 | 0.0006±0.0002 | 0.09±0.03 |
| A_2_-*Numt15* | 11,690 | 11,690 | chr12 | 0.0003±0.0002 | 0.04±0.03 |
| A_2_-*Numt16* | 14,100 | 14,098 | chr13 | 0.0001±0.0001 | 0.01±0.01 |

Note: ^a^ sixteen *numts* represent the largest mitochondrial fragments transferred into the nuclear chromosomes in *G. arboreum*.
