# Supplementary material for: Rapid evolutionary divergence of diploid and allotetraploid Gossypium mitochondrial genomes
Source: BMC Genomics. 2017 Nov 13;18:876. doi: 10.1186/s12864-017-4282-5 (PMC5683544; doi:10.1186/s12864-017-4282-5)
Supplement: Supplementary file 7 — Nucleotide distances and divergence time (MYA) between mitochondrial sequences and corresponding numts in G. hirsutum. Note: a fifteen numts represent the largest mitochondrial fragments transferred into the nuclear chromosomes in G. hirsutum. b represents five fragments from nearly full length mitochondrial fragments transferred into the nuclear A03 chromosomes in G. hirsutum (Fig. 7C). (DOCX 17 kb) [file 12864_2017_4282_MOESM7_ESM.docx]

Table S4. Nucleotide distances and divergence time (MYA) between mitochondrial sequences and corresponding *numts* in *G. hirsutum*

| Larger *NUMT*s^a^ | Length (bp) in Mitogenome | Length (bp) in Chromosome | Distribution in Chromosome | p-distance±SE | Divergence time (MYA) |
| --- | --- | --- | --- | --- | --- |
| AD_1_-*Numt1* | 6,965 | 6,904 | A01 | 0.0262±0.0018 | 3.91±0.27 |
| AD_1_-*Numt2*^b^ | **10,548** | **10,548** | **A03** | **0.0031±0.0005** | **0.46±0.07** |
| AD_1_-*Numt3* ^b^ | **4,130** | **4,130** | **A03** | **0.0022±0.0008** | **0.33±0.12** |
| AD_1_-*Numt4*^b^ | **3,323** | **3,320** | **A03** | **0.0069±0.0014** | **1.03±0.21** |
| AD_1_-*Numt5* ^b^ | **2,428** | **2,419** | **A03** | **0.0025±0.0010** | **0.37±0.15** |
| AD_1_-*Numt6* ^b^ | **2,081** | **2,081** | **A03** | **0.0062±0.0017** | **0.93±0.25** |
| AD_1_-*Numt7* | 6,883 | 6,872 | A06 | 0.0162±0.0012 | 2.42±0.18 |
| AD_1_-*Numt8* | 14,706 | 14,648 | A08 | 0.0564±0.0018 | 8.42±0.27 |
| AD_1_-*Numt9* | 4,960 | 4,944 | A08 | 0.0625±0.0040 | 9.33±0.60 |
| AD_1_-*Numt10* | 4,691 | 4,682 | A08 | 0.0588±0.0037 | 8.78±0.55 |
| AD_1_-*Numt11* | 5,112 | 5,093 | D06 | 0.0061±0.0010 | 0.91±0.15 |
| AD_1_-*Numt12* | 5,977 | 5,972 | D06 | 0.0077±0.0013 | 1.15±0.19 |
| AD_1_-*Numt13* | 4,719 | 4,684 | D10 | 0.0521±0.0031 | 7.78±0.46 |
| AD_1_-*Numt14* | 5,770 | 5,731 | D10 | 0.0651±0.0032 | 9.72±0.48 |
| AD_1_-*Numt15* | 5,624 | 5,565 | D10 | 0.0766±0.0032 | 11.43±0.48 |

Note: ^a^ fifteen *numts* represent the largest mitochondrial fragments transferred into the nuclear chromosomes in *G. hirsutum*.

^b^ represents five fragments from nearly full length mitochondrial fragments transferred into the nuclear A03 chromosomes in *G. hirsutum* (Figure 7C).
