# Supplementary material for: Rapid evolutionary divergence of diploid and allotetraploid Gossypium mitochondrial genomes
Source: BMC Genomics. 2017 Nov 13;18:876. doi: 10.1186/s12864-017-4282-5 (PMC5683544; doi:10.1186/s12864-017-4282-5)
Supplement: Supplementary file 8 — Nucleotide distances and divergence time (MYA) between mitochondrial sequences and corresponding numts in G. barbadense. Note: a twelve numts represent the largest mitochondrial fragments transferred into the nuclear chromosomes in G. barbadense. (DOCX 16 kb) [file 12864_2017_4282_MOESM8_ESM.docx]

Table S5. Nucleotide distances and divergence time (MYA) between mitochondrial sequences and corresponding *numts* in *G. barbadense*.

| Larger *NUMT*s^a^ | Length (bp) in Mitogenome | Length (bp) in Chromosome | Distribution in Chromosome | p-distance±SE | Divergence time (MYA) |
| --- | --- | --- | --- | --- | --- |
| AD_2_-*Numt1* | 10,816 | 10,816 | A01 | 0.0002±0.0001 | 0.03±0.01 |
| AD_2_-*Numt2* | 8,909 | 8,909 | A02 | 0.0001±0.0001 | 0.01±0.01 |
| AD_2_-*Numt3* | 7,691 | 7,691 | A02 | 0.0000±0.0000 | 0.00±0.00 |
| AD_2_-*Numt4* | 7,476 | 7,476 | A06 | 0.0000±0.0000 | 0.00±0.00 |
| AD_2_-*Numt5* | 9,952 | 9,946 | A08 | 0.0569±0.0025 | 8.49±0.37 |
| AD_2_-*Numt6* | 9,633 | 9,634 | A08 | 0.0020±0.0005 | 0.30±0.07 |
| AD_2_-*Numt7* | 9,485 | 9,485 | A08 | 0.0001±0.0001 | 0.01±0.01 |
| AD_2_-*Numt8* | 7,361 | 7,357 | A08 | 0.0082±0.0009 | 1.22±0.13 |
| AD_2_-*Numt9* | 7,115 | 7,105 | A08 | 0.0180±0.0016 | 2.69±0.24 |
| AD_2_-*Numt10* | 6,569 | 6,569 | A08 | 0.0000±0.0000 | 0.00±0.00 |
| AD_2_-*Numt11* | 9,671 | 9,671 | A09 | 0.0004±0.0002 | 0.06±0.03 |
| AD_2_-*Numt12* | 6,546 | 6,546 | A11 | 0.0000±0.0000 | 0.00±0.00 |

Note: ^a^ twelve *numts* represent the largest mitochondrial fragments transferred into the nuclear chromosomes in *G. barbadense*.
